# Galectin-3 Inhibition Is Associated with Neuropathic Pain Attenuation after Peripheral Nerve Injury

**DOI:** 10.1371/journal.pone.0148792

**Published:** 2016-02-12

**Authors:** Zhicong Ma, Qi Han, Xiaolei Wang, Zisheng Ai, Yongjun Zheng

**Affiliations:** 1 Department of Anesthesiology, The 2nd Hospital of Shanxi Medical University, Taiyuan, 030001, China; 2 Department of Anesthesiology, Xiangya Hospitalaffiliated to Central South University, Changsha 410008, China; 3 Department of Pain Management and Shanghai Key Laboratory of Clinical Geriatric Medicine, Huadong Hospital affiliated to Fudan University, No.221, West Yan'an Road, Shanghai, 200040, China; 4 Department of Preventive Medicine, College of Medicine, Tongji University, Shanghai 200092, China; Uniformed Services University, UNITED STATES

## Abstract

Neuropathic pain remains a prevalent and persistent clinical problem because it is often poorly responsive to the currently used analgesics. It is very urgent to develop novel drugs to alleviate neuropathic pain. Galectin-3 (gal3) is a multifunctional protein belonging to the carbohydrate-ligand lectin family, which is expressed by different cells. Emerging studies showed that gal3 elicits a pro-inflammatory response by recruiting and activating lymphocytes, macrophages and microglia. In the study we investigated whether gal3 inhibition could suppress neuroinflammation and alleviate neuropathic pain following peripheral nerve injury. We found that L5 spinal nerve ligation (SNL) increases the expression of gal3 in dorsal root ganglions at the mRNA and protein level. Intrathecal administration of modified citrus pectin (MCP), a gal3 inhibitor, reduces gal3 expression in dorsal root ganglions. MCP treatment also inhibits SNL-induced gal3 expression in primary rat microglia. SNL results in an increased activation of autophagy that contributes to microglial activation and subsequent inflammatory response. Intrathecal administration of MCP significantly suppresses SNL-induced autophagy activation. MCP also inhibits lipopolysaccharide (LPS)-induced autophagy in cultured microglia *in vitro*. MCP further decreases LPS-induced expression of proinflammatory mediators including IL-1β, TNF-α and IL-6 by regulating autophagy. Intrathecal administration of MCP results in adecreased mechanical and cold hypersensitivity following SNL. These results demonstrated that gal3 inhibition is associated with the suppression of SNL-induced inflammatory process andneurophathic pain attenuation.

## Introduction

Peripheral nerve injury (PNI) often results in chronic neuropathic pain and affects approximately 3–8% of people worldwide [1]. Neuropathic pain, associated with local neuroinflammation in the spinal cord, is a severe incapacitating condition that adversely affects the quality of life of sufferers [2–3].The advances in suitable therapy for the purpose of decreasing neuropathicpain have been limited because the pathophysiological mechanism causing this remains unclear.

Microglial cells are the resident immune cells of thecentral nervous system [4]. Following PNI microglial cells accumulate within the spinal cord and adopt a proinflammatory phenotype which contributes to neuropathic pain [5–6]. Dysregulation of cytokines implicated in a variety of painful neurological diseases in animals and in some clinical neuropathic pain conditions [3, 7]. In mouse model of nerve injury, tumor necrosis factor (TNF) and IL-1β expression level are significantly upregulated as early as 1 hour after chronic constriction injury in injured sciatic nerves and dorsal root ganglion [8]. Pro-inflammatory mediators IL-1β, TNF-α and iNOS are overexpressed in rats subjected to L5 spinal nerve ligation (SNL) [9]. In patients with complex regional pain syndrome, the mRNA levels of pro-inflammatory cytokines TNF and IL-2 are increased, whereas anti-inflammatory cytokines IL-4 and IL-10 levels are reduced [10].

Galectin-3 (gal3), a member of the β-galactoside-binding lectin family [11], is a multifunctional protein with various biological functions, including the cancer cell proliferation and invasion, and angiogenesis [12–14]. Recent studies showed that gal3 is involved in the inflammatory response, and its expression is upregulated in microglia after ischemic injury [15–17]. After exposure to gal3, glial cells produce high levels of proinflammatory mediators and exhibite activated properties [18].Gal3 knockout significantly reduces microglial activation and induces 4-fold decrease in microglia proliferation [16]. Emerging studies showed that modified citrus pectin (MCP), a derivative of pectin, can bind to the gal3 carbohydrate recognition domain thereby predominantly antagonising functions linked to this role [19]. For example, MCP could reduce gal3 expression and disease severity in experimental acute kidney injury [19].Pharmacological inhibition of gal3 by MCP markedly prevents aldosterone-induced cardiac and renal fibrosis [20].

Burguillos *et al* demonstrated that gal3 acts as an endogenous toll-like receptor (TLR)-4ligand, and can initiate a TLR4-dependent inflammatory response in microglia in a murine neuroinflammatory model and in human stroke subjects [15]. Gal3-dependent-TLR4 activation contributes to sustained microglia activation, prolonging the inflammatory response in the brain. TLR4 is a sensor for autophagy associated with inflammatory response [21]. TLR4-induced autophagy contributes to microglial activation and inflammatory injury and might provide novel therapeutic interventions for intracerebral haemorrhage (ICH) [22]. Autophagy is an evolutionarily conserved, lysosome-mediated intracellular catabolic process by which cells remove their damaged organelles and long-lived proteins for the maintenance of cellular homeostasis [23–24]. Autophagy plays an important role in both innate and adaptive immune responses. Autophagy activation in microglia is closely linked with neuroinflammation. Guo*et al* reported that cocaine elicits an increased expression and release of inflammatory factors (TNF, IL1B, IL6, and CCL2) in microglia by increasing the activation of autophagy [25]. Targeting autophagic proteins could be considered as a therapeutic strategy for the treatment of cocaine-related neuroinflammation diseases.

Based on above findings, we speculated whether gal3 inhibition by MCP protects against neuroinflammation and pain hypersensitivity by reducing the activation of autophagy. Our results revealed the therapeutic effects of MCP on SNL-induced neuropathic pain in rats.

## Materials and Methods

### 2.1 Animals and L5 spinal nerve ligation (SNL)

All experiments were performed according to the guidelines of the International Association for the Study of Pain and were approved by the Animal Care and Use Committee of Fudan University. Male Sprague-Dawley rats of body weight ranging from (180–220 g) were obtained from the Chinese Academy of Sciences (Shanghai, China). These animals were housed in a controlled environment with free access to food and water. Every effort was made to minimize pain and suffering, and the number of rats used was the least required to obtain significant statistical power.

The L5 SNL model was produced as previously described [26–27]. Briefly, rats were anesthetized with isoflurane (2.5%, Baxter, Deerfield, IL), and an approximately 2-cm long skin incision was made along the rat’s back. After removal of L6 transverse spinal process, the L5 spinal nerve was identified and ligated tightly with a 3–0 silk thread without damage to the dorsal root ganglion or other nerves. In sham-operated rats, the left L5 spinal nerve was isolated, without ligation. In each group, the ipsilateral L4 spinal nerve remained untouched, and the right side was not subjected to any surgery.

### 2.2 Modified citrus pectin (MCP)

pH and temperature modification of pectin was carried out as previously described[28]. Briefly, pectin from citrus peel (Sigma Aldrich, CA, USA) was dissolved in distilled water at a concentration of 1.5% at 60°C and the pH was adjusted to approx. 10.0 with NaOH. The solution was then cooled to room temperature while adjusting the pH to 3.0. Insoluble material was pelleted, and the supernatant was stored overnight at room temperature. The pH was next adjusted to 6.3, and the MCP was precipitated with 9 volumes of absolute ethanol and frozen at −20°C. The precipitate was filtered, washed with acetone on Whatman filters and then dried in vacuo. The doses of MCP (1 μg/μl) were chosen on the basis of previous studies [28] and pilot experiments performed in our laboratory.

### 2.3Intrathecal injections

Intrathecal administration of MCP was performed by lumbar puncture described by Calvo*et al* [5]. Under anaesthesia a 26G-gauge needle was inserted between the L5 and L6 vertebrae. About 20μL of working solution containing MCP (100 mg/kg/day, n = 10), which was referred to as Calvier’s study [29] and according to our preliminary results, was given once a day for 2 weeks. Saline was used for control injections (SNL group, n = 10). The quality of each injection was ensured by the observation of an injection-induced tail-flick.

### 2.4 Primary microglia cultures

Primary mixed microglial cultures were prepared as described previously [9, 30]. Briefly, four rats were sacrificed and lumbar enlargements of spinal cord were rapidly removed. Spinal cords were then ground in 4 ml ice-cold Hanks’ balanced salt solution. Suspensions were passed through a 70-μm cell strainer and cells were centrifuged at 400g for 10 min. A density gradient consisting of 4 ml cells in 75% Percoll, 3 ml 50% Percoll, 3 ml 35% Percoll, and 2 ml PBS was centrifuged (1000g for 20 min, at 10°C). Cells at the 50/75% interface were collected, washed in ice-cold PBS, and maintained in DMEM with10% FBS (Gibco, Carlsbad, CA).

### 2.5 ELISA assay

IL-1β, TNF-α and IL-6 released into the culture medium were assessed using ELISA kits following stimulation with LPS (0.5ng/μl) for 24 haccording to the manufacture’s instruction.ELISA kits were purchased from R&D system (MN, USA). Plates were read using a microplate reader (Model 550, Bio-Rad, USA) at a 450 nm wavelength.

### 2.6 Quantitative real-time PCR (qPCR)

Total RNA was extracted from the fifth lumbar dorsal horns ipsilateral to SNL or cultured microglia using Trizol reagent (Invitrogen, Carlsbad, CA).The reverse transcription (RT) for mRNA was carried out using the OligodT primer. qPCR was performed on Applied Biosystems 7300 real-time PCR system (Applied Biosystems, Foster City, CA) using a standard protocol from the SYBR Green PCR kit (Toyobo, Osaka, Japan). The qPCR primers for gal3 are as follows: gal3 forward (TGTGCCTTATAACCTGCCTTTGC) and gal3 reverse (AACCGACTGTCTTTCTTCCCTTC). β-actin was used as references for mRNAs. Each sample was analyzed in triplicate. The 2^-ΔΔCt^ method was used to quantify the relative levels of gene expression.

### 2.7 Behavior Testing

Mechanical sensitivity was tested using calibrated von Frey filaments (Stoelting, Wood Dale, IL) applied to the plantar surface of the hind paw ipsilateral to surgery. A servo-controlled mechanical stimulus was applied to the plantar surface at 10-min intervals. The withdrawal threshold of each paw was tested three times for each time point and mean values were used in the analysis. A maximum cutoff of 50g was used. To test the cold allodynia, a drop (50 μl) of acetone was applied to the centre of the plantar face of a hindpaw. Acetone was applied alternately twice to each hindpaw, with 5-min between each successive application. Responses were monitored during 2 min after acetone application.

### 2.8 Western blot analysis

The fifth lumbar dorsal horns ipsilateral to SNL or sham surgery were collected after behavioral tests and homogenized with ice-cold lysis buffer (50 mMTris-HCl, 1 mM EDTA, 0.1% SDS, 150 mMNaCl, 1% Igepal CA-630, 50 mMNaF, and 1 mM NaVO3) containing a mixture of protein inhibitors (Sigma) including PMSF (2 mM), leupeptin (10 μg/ml) as well as aprotinin (10 μg/ml).Western blot analysis to assess gal3 protein expression was performed as previously described [31]. The anti-gal3 primary antibodies were purchased from Abcam (ab31707). Goat anti-rabbit secondary antibody was obtained from Santa Cruz Biotechnology (Santa Cruz, CA). β-actin primary antibodies were purchased from Sigma (MO, USA).

### 2.9 Fluorescence microscopy analysis

Microglial cells isolated from ratswere seeded on poly-L-lysine-coated coverslips sitting on the bottom of 12-well plates. After 24 h, cells were rinsed once in phosphate-buffered saline (PBS) and fixed with 4% paraformaldehyde, followed by blocking in a solution containing 5% BSA(Sigma) containing 0.1% Triton X-100(Sigma) for 1 h at room temperature. Cells were then incubated overnight at 4°C with the indicated primary antibody (gal3: 1μg/ml, ab31707, Abcam; Iba1: 1:1000, ab107159, Abcam). The coverslips were washed with 0.1 M PBS and incubated with FITC-conjugated goat anti-rabbit IgG (1:100, Chemicon, USA), or Cy3-conjugated rabbit anti-goatIgG (1:100, Chemicon, USA) for 1 h at room temperature. Fluorescentimages were obtained with the FluoView™ FV1000 confocal microscope (Olympus, Tokyo, Japan).

Autophagy assay was carried out according to the manufacturers' instructions (ENZ-51031-K200,Enzo life science). Briefly, microglial cells were seeded into 12 well plates (1–2 × 10^4^ cells/well). Following compound treatment, cells were washed once in PBS and resuspended in 1×Cyto-ID Green autophagy detection reagent and incubated at room temperature for 30 min. Analysis of the stained cells was performed by wide-field fluorescence(Olympus, Tokyo, Japan).Use a standard FITC filter set for imaging the autophagic signal.

### 2.10 Statistics

Data are presented as mean ± standard deviation (SD) from at least three separate experiments. The unpaired Student *t* test was used to assess statistical differences between 2 experimental conditions. Differences among more than 2 experimental conditions were tested by the one-way ANOVA, followed by the Scheffé test to analyze differences between groups. Behavioural data was analyzed using RM two-way ANOVA. Differences were deemed statistically significant at *p*< 0.05.

## Results

### 3.1 MCP suppresses SNL-induced upregulation of gal3

**Fig 1:**
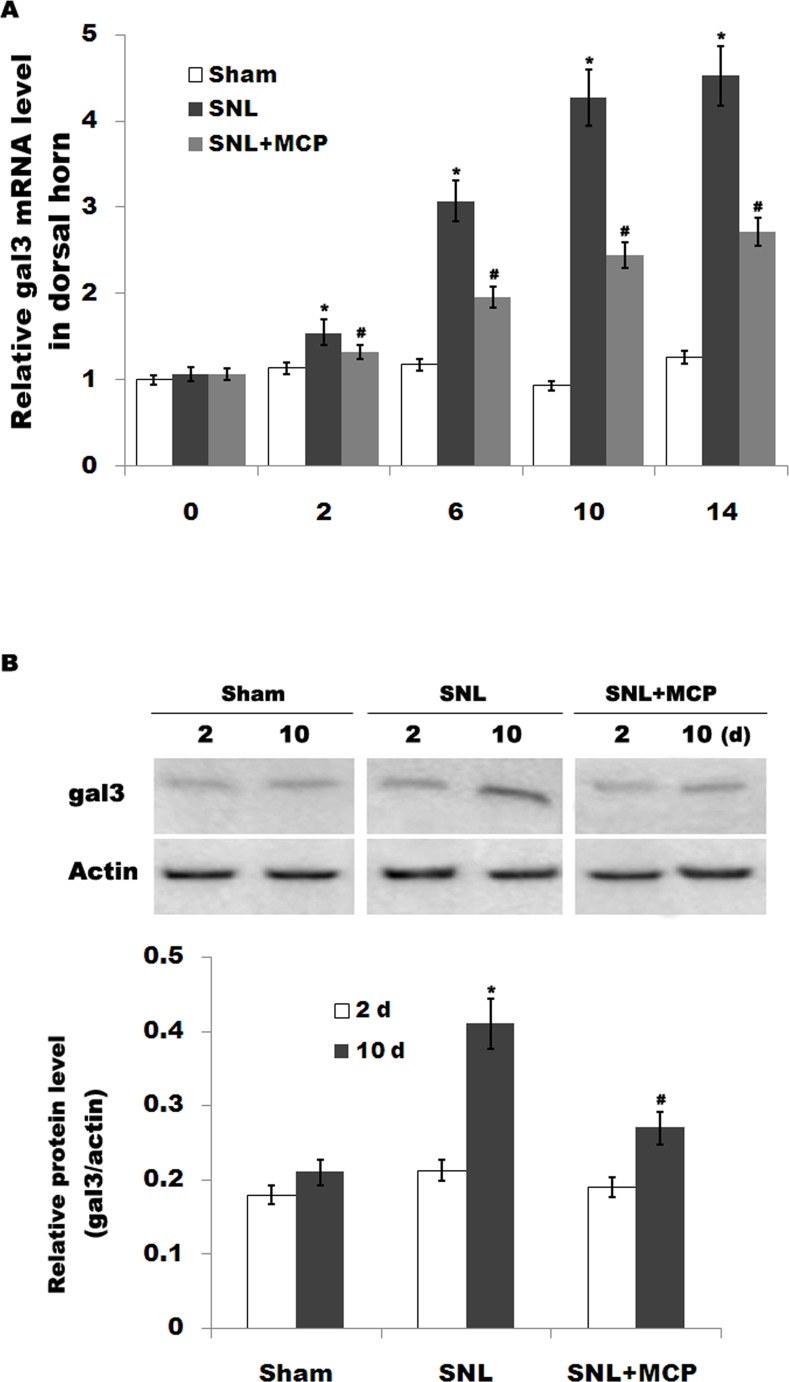
Gal3 expression in DRGs is increased after SNL. (A) The mRNA level of gal3 is increased in DRGs of rats subjected to L5 SNL. Total RNA was extracted from the fifth lumbar dorsal horn sections and was subjected to real-time PCR to analyze the relative expression level of gal3 in each sample. Each sample was analyzed in triplicate. The 2^-ΔΔCt^ method was used to quantify the relative levels of gal3. β-actin was used as reference for mRNA. n = 10. *p< 0.05 vs sham, # *p*< 0.05 vs SNL group. (B and C) Western blot analysis of gal3 protein expression in the fifth lumbar dorsal horn sections in each sample.*p< 0.05 vs sham, # *p*< 0.05 vs SNL group.

**Fig 2:**
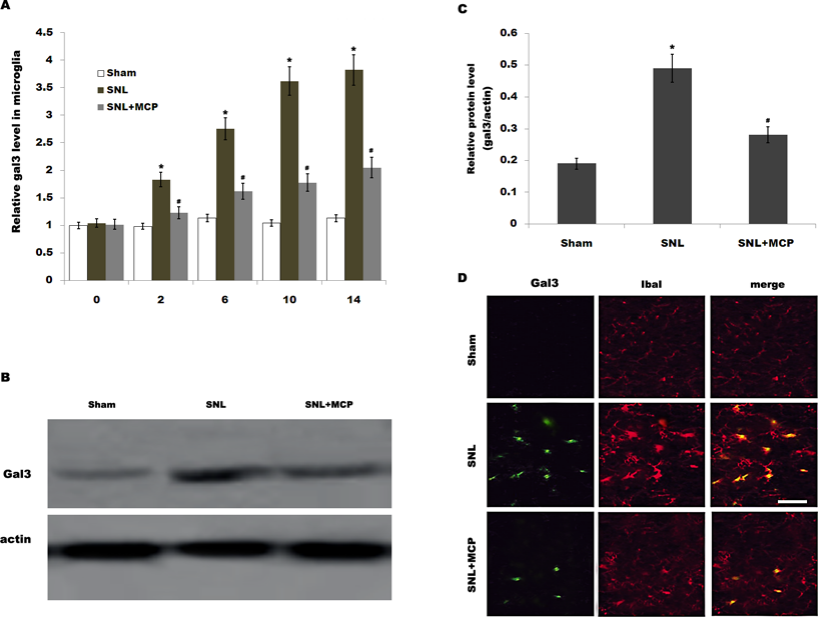
Gal3expression in microglia is increased after SNL. (A) Primary spinal microglial cells were isolated from sham-, SNL-, and MCP-treated rats at the indicated time, and relative gal3 mRNA levels were analyzed by real-time PCR according to the methods described above. n = 10. *p< 0.05 vs sham, # *p*< 0.05 vs SNL group. (B and C) Primary spinal microglial cells were isolated from sham-, SNL-, and MCP-treated rats at the indicated time, and relative gal3protein levels were analyzed usingwestern blot. n = 10. *p< 0.05 vs sham, # *p*< 0.05 vs SNL group. (D) Double immunofluorescence labelling for gal3 (green) and cell-type markers (Iba-1, microglia marker, red) in fifth lumbar dorsal horn sections 10 d after peripheral nerve injury. Scale bar = 50 μm.

Gal3 is involved with microglial activation and subsequent inflammatory response in brain ischemia and in neurodegenerative disorders. However, the role of gal3 in the regulation of neuropathic pain following SNL remains unclear. Here we first assayed whether L5 SNL changes the expression pattern of gal3. As shown in Fig 1A,gal3 mRNA level is promptly upregulated in DRGs after L5 SNL and maintained at higher values throughout a period of 14 d compared to that for the sham-operated group. Similarly, gal3 protein expression in DRGs is also increased after SNL compared with control (Fig 1B). Intrathecal administration of MCP, a gal3 inhibitor, markedly inhibits gal3 expression in DRGs at the mRNA and protein level (Fig 1A and 1B). We further analyzed the expression level of gal3 in primary microglial cells because microglial cells are key cellular mediators of neuroinflammatory processes and neuropathic pain. Fig 2A showed that the mRNA level of gal3, which is low in sham-operated rats, is increased in ipsilateral spinal microglia after L5 SNL, starting from postoperative 2 d, and persisting a period of 14 d. Western blot analysis showed that the protein level of gal3 is also upregulated following L5 SNL (Fig 2B and 2C). We next performed double-immunolabeling with cell type specific markers and found that almost all gal3+ cells are positive for the microglial markers Iba1, and spinal microglia with the high levels of gal3also indicates an activated morphology (Fig 2D). Intrathecal administration of MCP significantly suppresses gal3 expression in spinal microglia at the mRNA and protein level (Fig 2A–2D). These data suggest that MCP treatment could reduce L5 SNL-induced upregulation of gal3 in rats.

### 3.2 MCP inhibits SNL-induced activation of autophagy in spinal microglia

**Fig 3:**
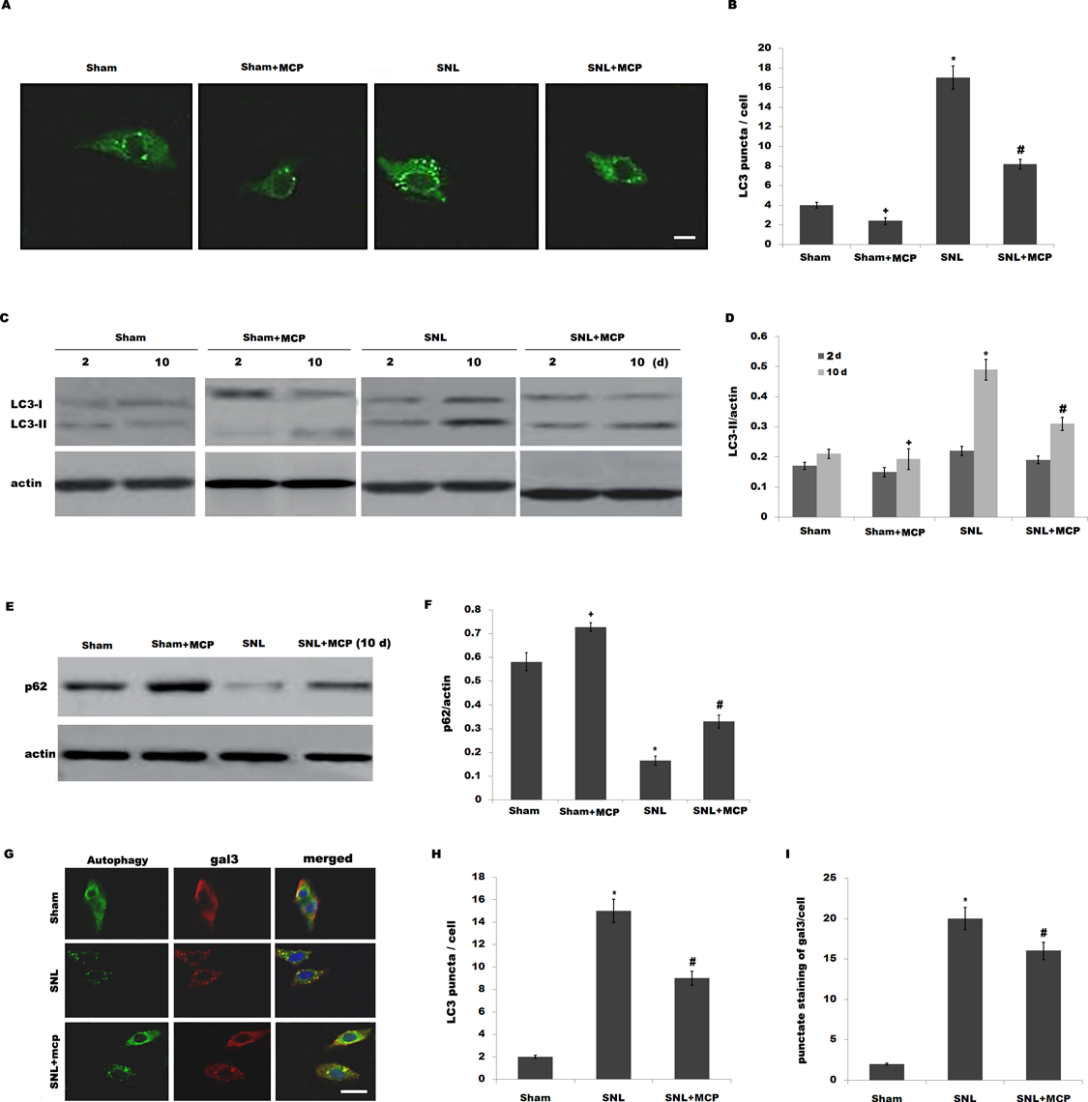
MCP inhibits SNL-induced activation of autophagy in spinal microglia. (A and B) Primary spinal microglial cells were isolatedfrom sham-, SNL-, and MCP-treated rats at day 10, and the autophagosome formation was visualized by assaying LC3 green puncta. Punctate staining is indicative for the redistribution of LC3 to autophagosomes. The average number of LC3 green puncta per cell with standard deviation for each group is presented. *p< 0.05 vs sham, # *p*< 0.05 vs SNL group. (C and D) Primary spinal microglial cells were isolated from sham-, SNL-, and MCP-treated rats at the indicated time, and LC3B protein levels were assayed using western blot analysis. *p*< 0.05 vs sham, # *p*< 0.05 vs SNL group. (E and F) Primary spinal microglial cells were isolated from sham-, SNL-, and MCP-treated rats at day 10, and the protein levels of p62 were assayed using western blot analysis. *p*< 0.05 vs sham, # *p*< 0.05 vs SNL group. (G-I) Microglial cells were isolated from sham-, SNL-, and MCP-treated rats at day 10, and the expression level of gal3 and autophagy activation was assayed using double-label immunofluorescence analysis. Scale bar = 50 μm.

Burguillos *et al* demonstrated that microglia-secreted gal3 acts as a toll-like receptor (TLR)-4 ligand and promotes microglial activation in the brain [15]. In addition, emerging studies revealed that TLR4 is a sensor for autophagy, and TLR4-mediated activation of autophagy contributes to microglial activation and pro-inflammatory injury in intracerebral haemorrhage [22]. Therefore we speculated whether L5 SNL induces the activation of autophagy and MCP treatment inhibits SNL-induced autophagy. Primary microglia cells were isolated from sham-, SNL-, and MCP-treated rats, and respective activation of autophagy was analyzed. Fig 3A–3D showed that following SNL, there is an increase of LC3 green puncta representing autophagic vacuoles and an accumulation of LC3-II in spinal microgial cells, indicating that autophagy is activated. The autophagy flux was further determined by assaying the decrease of p62/SQSTM1 protein level, a well-established autophagy substrate (Fig 3E and 3F). Intrathecal administration of MCP markedly inhibits SNL-induced autophagy (Fig 3A–3D). Fig 3E and 3F showed that MCP significantly suppresses SNL-induced decrease of p62 protein level. The expression level of gal3 and autophagy activation was further assayed by double-label immunofluorescence analysis. As shown in Fig 3G–3I, upregulated expression of gal3 by SNL is accompanied by increased punctuate of LC3, whereas MCP treatment decreases the punctate staining of gal3 and inhibits autophagy activation.

**Fig 4:**
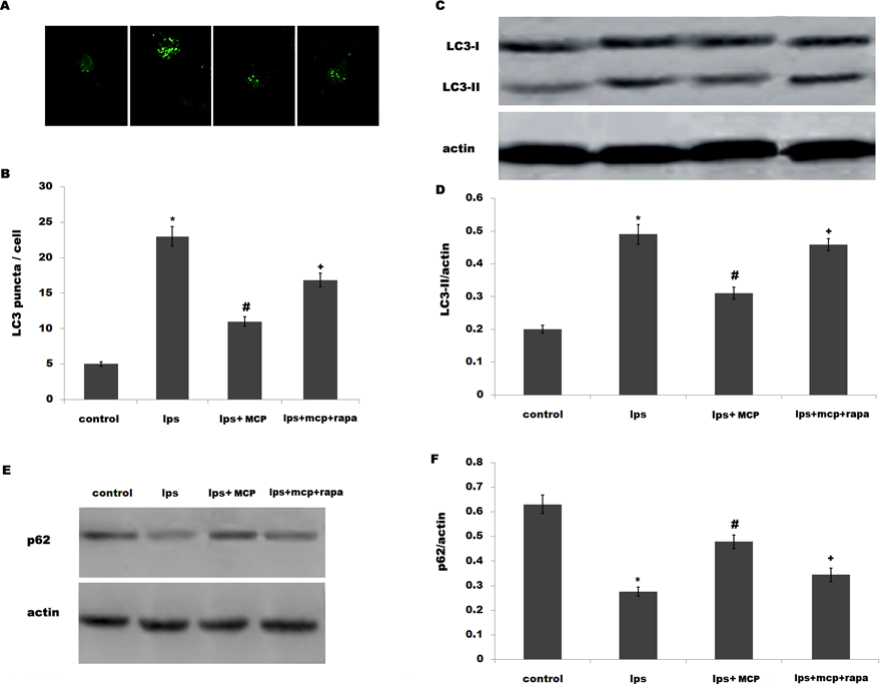
MCP inhibits LPS-induced activation of autophagy in microglia. (A and B) Mixed microglial cultures isolated from sham rats were treated with LPS (0.5ng/μl) and MCP (1 μg/μl), and the autophagosome formation was visualized by assaying LC3 green puncta. The average number of LC3 green puncta per cell with standard deviation for each group is presented. *p< 0.05 vs control, # *p*< 0.05 vs LPS group. Mixed microglial cultures isolated from sham rats were treated with LPS (0.5ng/μl) MCP (1 μg/μl) or Rapa (500 nM), and LC3B protein levels (C and D) or p62 protein levels (E and F) were assayed using western blot analysis. *p< 0.05 vs control, # *p*< 0.05 vs LPS group.

We then investigated the effect of MCP on the autophagy *in vitro*. Primary microglial cells were obtained from sham-operated rats and treated with MCP, and the activation of autophagy was analyzed. Lipopolysaccharide (LPS) is a known TLR4 ligand and could effectively activate autophagy in human and rat cells [32–33]. Here we treated microglial cells with LPS and MCP, and investigated the effect of MCP on autophagy in cultured microglia. After treatment with LPS, there is an increase of LC3 green puncta representing autophagic vacuoles and an accumulation of LC3-II in microglia, indicating that LPS activates autophagy (Fig 4A–4D). However, MCP treatment markedly inhibits LPS-induced autophagy activation (Fig 4A–4D). The autophagy flux was also demonstrated by assaying the protein level of p62/SQSTM1. Fig 4E and 4F showed that MCP significantly inhibits LPS-induced decrease of p62 protein level. Expectedly, rapamycin (Rapa) blocks the MCP inhibition of LPS-stimulated autophagy in microglia (Fig 4).

### 3.3 MCP inhibits neuroinflammation, and mechanicaland cold hypersensitivity after SNL

**Fig 5:**
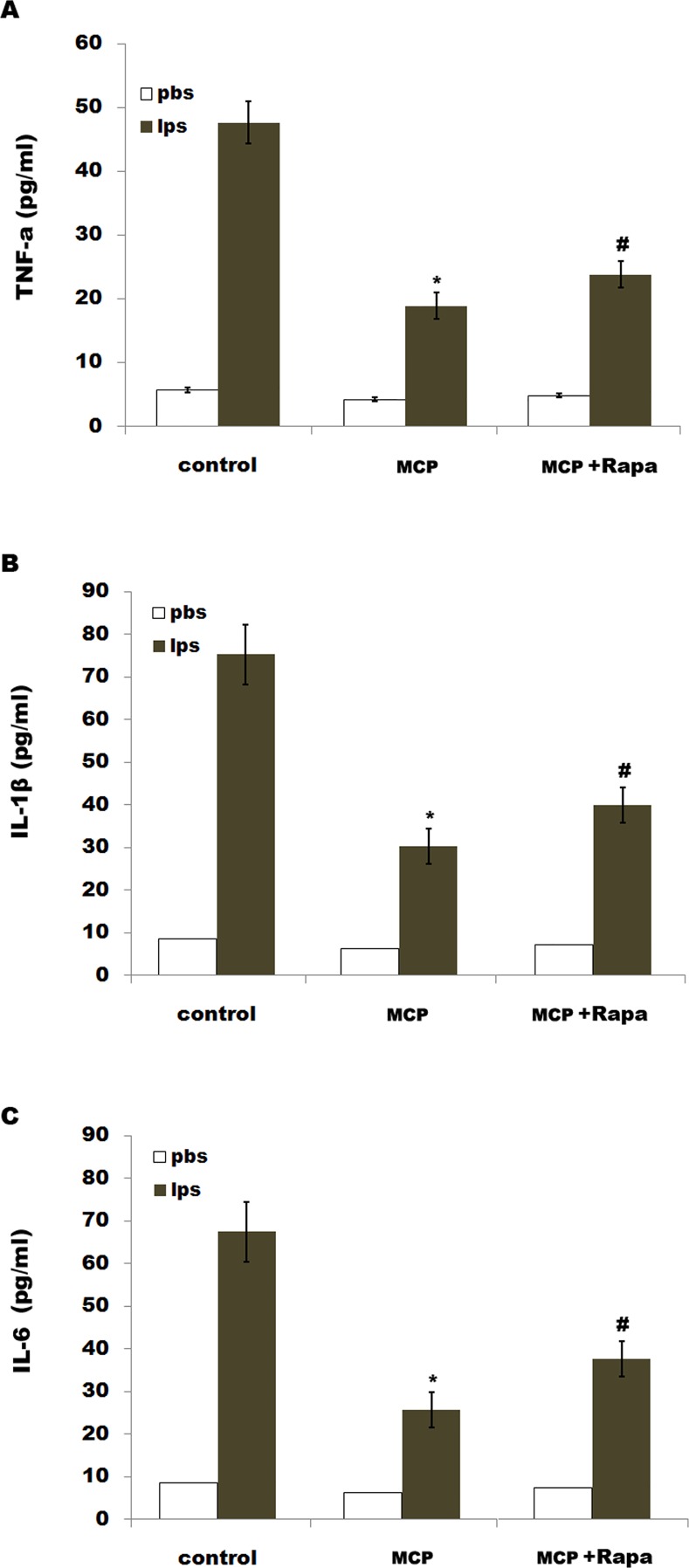
MCP inhibits LPS-induced releases of proinflammatory cytokines by regulating autophagy in microglia. Mixed microglial cultures isolated from sham rats were treated with LPS (0.5ng/μl), MCP (1 μg/μl) and Rapa (500 nM). ELISA analysis of TNF-α (A), IL-1β (B) and IL-6 (C) of protein levels were carried out using Elisa kit. Data are expressed as mean ± SD. *p< 0.05 vs control, # *p*< 0.05 vs MCP group.

To investigate the effect of gal3 inhibtion on regulating neuroinflammation and neuropathic pain following SNL, microglial cells were treated with MCP and proinflammatory cytokines levels were assessed after treatment with LPS. MCP significantly decreases LPS-induced expression of IL-1β, TNF-α and IL-6in spinal microglia (Fig 5A–5C). Rapa (a known inducer of autophagy) treatment reduces the effect of MCP on suppressing the release of pro-inflammatory cytokines (Fig 5A–5C).

**Fig 6:**
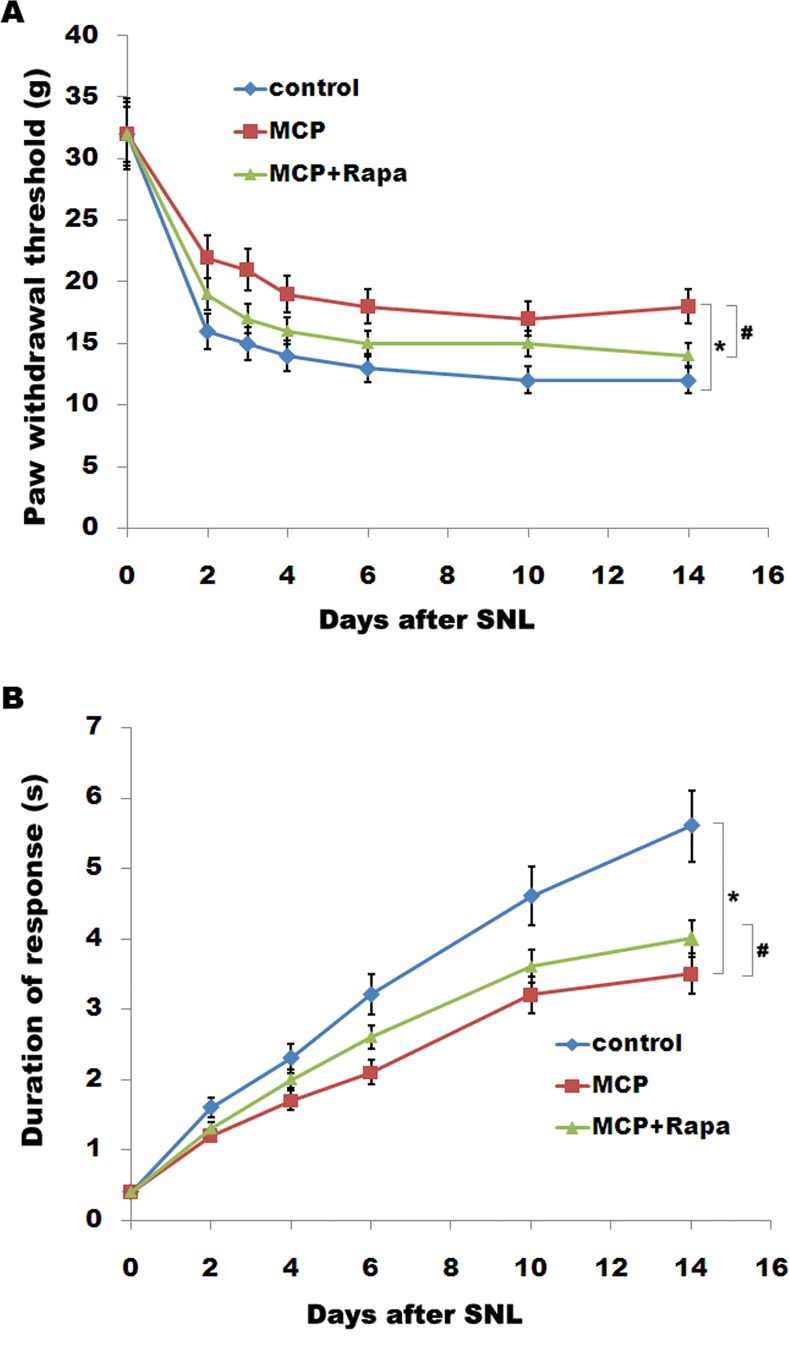
MCP results in a decreased mechanicaland cold hypersensitivity. Mechanical (B) and cold (C) pain-related hypersensitivity developed after treatment with MCP (100 mg/kg/day) and Rapa (1 mg/kg/day) at the indicated time after surgery. n = 8. Data are expressed as mean ± SD. *p< 0.05 vs control, # *p*< 0.05 vs MCP group.

To examine the role of gal3 inhibition in regulating mechanical sensitivity, we measured paw mechanical withdrawal threshold and cold allodynia after intrathecal administration of MCP. MCP treatment displays a marked increase in the paw mechanical withdrawal threshold in rats (Fig 6A). Cold hypersensitivity is also significantly different compared with control in rats (Fig 6B). Expectedly, Rapa treatment reduces the effect of MCP on suppressing pain hypersensitivity (Fig 6A and 6B). These results suggest that gal3 inhibition could effectively inhibit SNL-induced neuroinflammation and neuropathic pain, at least in part by regulating autophagy in rats.

## Discussion

Neuroinflammation is an early event associated with onset and progression of central sensitization and pain sensitivity. Glial cells, especially microglia, are a major central nervous system population that can modulate neuroinflammation[34]. Following PNI microglial cells accumulate within the spinal cord, and activated microglia adopt a pro-inflammatory phenotype [5]. Emerging evidence suggests that gal3 is involved in fine-tuning of the inflammatory responses at the periphery. For example, gal3 plays an important role in resident microglia activation and proliferation in response to ischemic injury [16].Gal3-induced activation of TLR4 signaling contributes to sustained microglia activation, prolonging the inflammatory response [15].Knockout of the gal3 gene represses microglia activation and induces marked decrease in microglia proliferation, which results in a significant increase in the size of ischemic lesion [16]. In our study we demonstrated that SNL induces an increased expression of gal3 in microglia, and gal3 inhibition by MCP decreases LPS-induced releases of IL-1β, TNF-α and IL-6. We further assayed the role of gal3 in regulating tactile allodynia. Gal3 inhibition by MCP results in a decreased mechanical and cold hypersensitivity in rats following SNL.

Autophagy, an intracellular degradation and energy recycling mechanism, is emerging as an important regulator of immune responses, and defects in autophagy have been linked to several inflammation-related diseases [23–24]. The connections between autophagy and inflammation are complex. On one hand, several studies demonstrated that autophagy negatively regulates inflammation to prevent the harmful amplification of inflammatory factors [35]. For example, inhibition of autophagy results in the activation of inflammasomes which control the proteolytic processing and secretion of IL -1β and IL-18 under inflammatory stress [36–37]. Also, inhibition of autophagy related16-like 1 (ATG16L1) increases the production of IL-1β and IL-18 in the mouse model of Crohn’s disease [38]. On another hand, many reports showed that autophagy contributes to microglial activation and inflammatory injury. Erythrocyte lysis induces TLR4-mediated microglial autophagy [22]. The autophagy inhibition suppresses microglial activation and inflammatory injury, and improves the neurological function after intracerebral haemorrhage [22].Also, cocaine treatment increases the expression of autophagy-related genes in brains, and upregulated autophagy contributes to cocaine-mediated activation of microglia. Inhibition of autophagy leads to a decreased expression and release of inflammatory factors (IL-1B, IL-6, and CCL2) in microglial cells[25]. In our study, we found that L5 SNL induces the activation of autophagy. Intrathecal administration of MCP reduces SNL-induced autophagy.MCP treatmen talso suppresses LPS-induced autophagy activation and decreases LPS-induced expression of IL-1β, TNF-α and IL-6 in spinal microglia. Rapa partially abrogates the effect of MCP on suppressing the release of pro-inflammatory cytokines. Finally, we assayed the effect of gal3 inhibitor in regulating tactile allodynia. MCP treatment results in a decreased mechanical and cold hypersensitivity in rats following SNL, at least in part by regulating autophagy. ***Conclusion*:** Our data demonstrated that MCP could effectively inhibit gal3 expression and SNL-induced neuroinflammation and neuropathic pain in rats, and suggest its candidacy as a new target for clinical management of peripheral nerve injury-induced neuropathic pain.
